# A semi-supervised approach for extracting TCM clinical terms based on feature words

**DOI:** 10.1186/s12911-020-1108-1

**Published:** 2020-07-09

**Authors:** Liangliang Liu, Xiaojing Wu, Hui Liu, Xinyu Cao, Haitao Wang, Hongwei Zhou, Qi Xie

**Affiliations:** 1 School of Statistics and Information, Shanghai University of International Business and Economics, Shanghai, 201620 China; 2 China National Institute of Standardization, Beijing, China; 3 Institute of Basic Research in Clinical Medicine, China Academy of Chinese Medical Sciences, Beijing, 100700 China; 4 Department of Academic Management, China Academy of Chinese Medical Sciences, Beijing, 100700 China

**Keywords:** TCM, NER, Clinical terms, Deep learning, Semi-supervised

## Abstract

**Background:**

A semi-supervised model is proposed for extracting clinical terms of Traditional Chinese Medicine using feature words.

**Methods:**

The extraction model is based on BiLSTM-CRF and combined with semi-supervised learning and feature word set, which reduces the cost of manual annotation and leverage extraction results.

**Results:**

Experiment results show that the proposed model improves the extraction of five types of TCM clinical terms, including traditional Chinese medicine, symptoms, patterns, diseases and formulas. The best F1-value of the experiment reaches 78.70% on the test dataset.

**Conclusions:**

This method can reduce the cost of manual labeling and improve the result in the NER research of TCM clinical terms.

## Background

Named entity recognition (NER) is an important research work in natural language processing. In the field of Traditional Chinese Medicine (TCM), there is a vast amount of ancient books and medical records, which contain a huge multitude of TCM clinical terms. These terms contain rich and high-value information. There are three main research significances for the TCM NER research [1]. Firstly, it is important to summarize the TCM clinical diagnosis and treatment rules. Since the TCM clinical corpus contains great amounts of information that include patient health, symptoms, diseases, and treatment plans based on clinical practices. Secondly, it benefits the construction of TCM expert systems, TCM knowledge graphs, and TCM QA systems [2]. Thirdly, the study of extracting TCM clinical terms promotes the standardization system construction of TCM clinical terms and help to make better comparison between TCM clinical terms and Modern clinical terms [3].

However, ancient Chinese language is extensively used in the TCM corpus which brings difficulties to TCM NER research [4]. In this paper, we introduce a semi-supervised approach for extracting TCM clinical terms based on feature words. In the experiments, five types of Chinese medicine clinical terms are automatically extracted from TCM related corpus, including: Chinese traditional medicines, formulas, diseases, patterns, and symptoms. The proposed method reduces the cost of manual labeling under the semi-supervised learning and improves the TCM clinical extraction ability based on the feature words. Results show that the proposed method can be used in related fields.

We mainly study from the BiLSTM-CRF [5] NER extraction model and made some improvements in the research. Firstly, we create the character vectors for the input layer from vectors trained from Wikipedia and TCM related corpus. Secondly, we propose a collection of TCM clinical term feature words and combine the clinical feature words with trained character vectors by adding a special length dimension. Thirdly, we use semi-supervised learning in model training for reducing the work of human feature annotation.

## Related work

NER is an important research content of information extraction. In the beginning, NER is mainly based on the linguistic knowledge. Many researchers primarily used the contextual information and internal components of terms [6–8]. They also combine linguistic knowledge with statistics to improve the result. The main statistical parameters include frequency, hypothesis testing, likelihood ratio, mutual information, etc. [9].

Afterwards, researchers employed the statistical model approach which transforms NER into a prediction problem. Common models used in NER are ME (Maximum Entropy), HMM (Hidden Markov Model) and CRF (Condition Random Field), etc. And such works can be reviewed by Nguyen et al. [10], and a large part of them are based on CRF [11, 12].

Recently, researchers began to apply deep learning models to NER, such as the CNN-CRF [13] and RNN-CRF [14, 15]. The main advantage of deep learning models is that in model training the researchers do not need to manually select features, as the model can learn task-specific representations, establishing different models based on the original information to achieve better NER results. For example, Peng et al. [16] and Ridel et al. [17] use the LSTM-CRF to get a better result in biomedical NER and German NER experiments. The main frameworks of these models are based on the combination of LSTM or CNN with CRF.

Another related area of our task is term extraction in the medical field, which is covered in biomedical science. Researchers adopted a dictionary-based approach for extraction, but with low precision [18, 19]. So, the researchers of biomedical NER gradually turned to statistical methods and their combinations to improve NER results. For example, Ahmed et al. [20] and Lei et al. [21] use SVM, KNN, DT, SVM to extract the named entities from biomedical corpus and Chinese clinical text. Shweta et al. [22] apply PSO (Particle Swarm Optimization) model for feature selection in NER research. Nowadays, the most popular methods have also been turned into using deep learning frameworks, including active learning [23], DNN (deep neural networks)-CRF [24] or LSTM-CRF [25–27].

The study of NER in Chinese clinical texts is based mainly on the DNN-CRF model [24]. However, in the TCM clinical corpus materials, the terms are mostly expressed in classical and semi-classical Chinese, which differs from the Chinese mandarin stylish clinical texts a lot. This is also the main obstacle in the TCM clinical terms extraction research. Therefore, in this paper we focus on the researches of TCM clinical terms NER in order to get a better NER extraction results. And that is why we introduce a semi-supervised approach for extracting TCM clinical terms based on feature words.

## Methods

### Datasets

In this paper, we use the TCM corpus *Formulas of Chinese Medicine* [28] as the main dataset for extracting the TCM clinical terms. The main reason to choose *Formulas of Chinese Medicine* is that it elaborates Chinese traditional formulas from various perspectives including traditional Chinese medicine, diseases, symptoms and patterns. It is a comprehensive description of the composition of the traditional Chinese medicine of the prescription for the TCM treatment. Specifically, this corpus describes the formulas with the pattern of prescriptions, the symptoms of prescription, and a variety of diseases that are treated accordingly. Therefore, in the *Formulas of Chinese Medicine*, the formula is the center, and the other four types of entities including symptoms, traditional Chinese medicine, patterns and diseases are used for the detailed description.

At the same time, we also refer to the national standard documents to ensure the reliability and correctness of the extracted entities. The documents are *National Standards for Clinical Diagnosis and Treatment of Traditional Chinese Medicine (Disease Part)* and *National Standards for Clinical Diagnosis and Treatment of Traditional Chinese Medicine (Certification Part).*

We divide the labeled dataset into three subsets as training, development and testing respectively. Meanwhile a large number of unlabeled datasets are also prepared for semi-supervised learning, from the TCM corpus *Formulas of Chinese Medicine* as well*.*

### Extraction models

The FCM corpus is firstly segmented and labeled with the named entities using “IOB” tagging sets, which is used to solve the basic problems in sequence labeling that frequently encountered in many NLP researches. Each character is labeled as “B-X” (the first character of a type X term), “I-X” (the second or following character of a type X term) or “O” (not in any terms). After the labeling work, the annotated corpus is divided into three parts: training set, development set and test set.

**Fig. 1:**
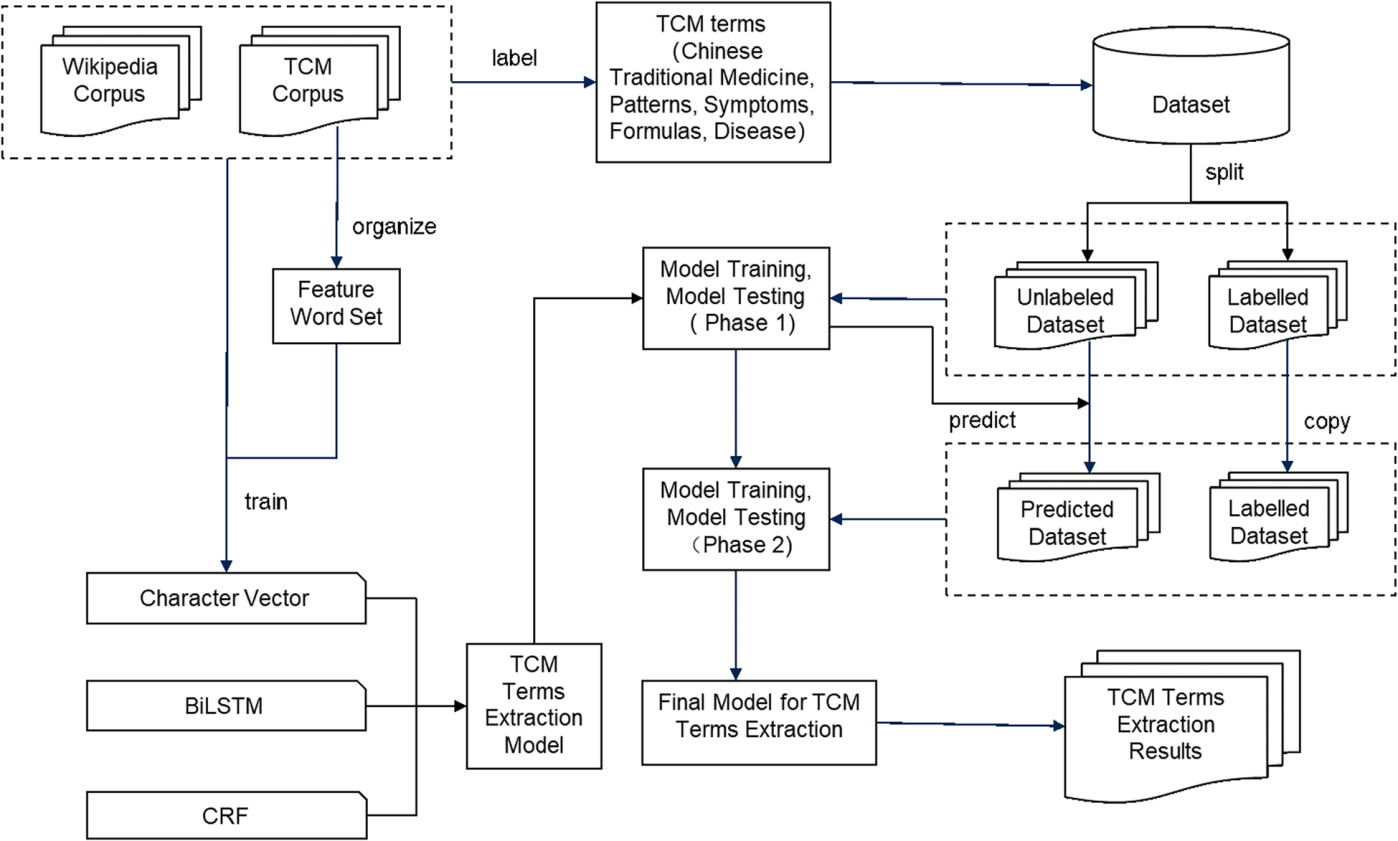
TCM Clinical Terms Extraction Model. This figure shows the overall TCM clinical terms extraction steps

The general framework of the extraction model is based on BiLSTM-CRF [5, 29, 30]. The input layer is for the character vectors corresponding to Chinese characters in the corpus [31, 32]. The second layer is for the BiLSTM, which is used to perform feature learning on the sequence, and the third layer is for the CRF, which helps to select the sequence result with the highest probability in the predicted sequence. Therefore, the task of extracting the TCM clinical terms is transformed into labeling whole sequence of the corpus using the deep learning models. An overview of the entire framework of the model is shown in Fig. 1.

### Character vector training

There are many works focusing on the character-level feature such as morphology, and the use of the character-level features on NNs (neural networks) such as CNNs [33]. The first step is to train the character vector in the following steps.

#### Training a vector using corpus related to TCM

We use the TCM corpus such as medical records, ancient Chinese medicine books to train the character vector. We denote the *i* th word with $Embedding\_C{M}_{w_i}$, and the whole size of the word set is *M*.

#### Training a vector using an encyclopedia corpus

We use the corpus such as *Baidu Baike* or *Wikipedia* to train another character vector. And we define this kind of vector as $Embedding\_{WIKI}_{w_i}$.

#### Combining the two vectors using customized weights

**Fig. 2:**
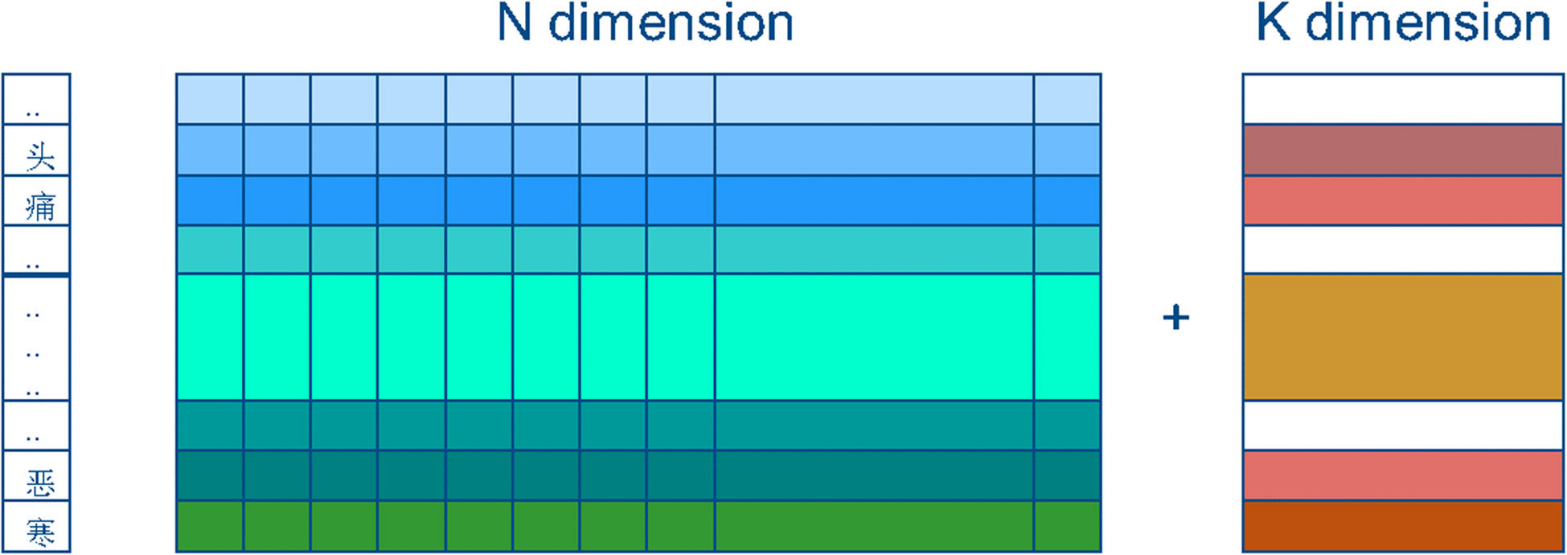
The example of the combined of two kinds of character vectors. We combine the character vectors using the illustrated method in the figure

We denote the custom weight as *λ* and use *λ* to combine the two trained character vectors to form the final character vector (Fig. 2). And be denoted the final character vector as $Embeddin{g}_{w_i}$. The following Eq. 1 shows the calculation of the final vector. In Eq. 1, the size of the word set is *M*, and *w*_*i*_ represents the i th word or character.

1 $$ {\displaystyle \begin{array}{c} Embeddin{g}_{w_i}=\lambda \cdotp Embedding\_C{M}_{w_i}+\left(1-\lambda \right)\cdotp Embedding\_{WIKI}_{w_i}\\ {}i=1,2,\dots, M;0\le \lambda \le 1\end{array}} $$

### Feature word set

The second step of the model is to construct a feature word set in the following steps:

#### Collecting the feature word sets

Collect and sort out the words whose frequency in the TCM clinical terms is greater than the set threshold *T* as the feature word, and add them into the feature word set table.

#### Combine two kinds of character vectors

Classify different types of feature words into categories, meanwhile denote the *j* th category of feature word as *P*_*j*_ . The character vectors of the feature words from each category are given a certain dimension, which is initialized by a Gaussian distribution, as shown in the following formula:

2 $$ Embeddin{g}_{w_i}=\Big\{{\displaystyle \begin{array}{c} Embeddin{g}_{w_i}+ Embedding\_ GAU{S}_{w_i},{w}_i\in Q\ \\ {} Embeddin{g}_{w_i}+ Embedding\_ ZERO,{w}_i\notin Q\end{array}}\operatorname{} $$

Where *Q* is the set of feature words. We define the relationship between *Q* and *P*_*j*_ as follows.

3 $$ \bigcup \limits_{j=1}^N{P}_j=Q $$

We define an indicator function ${X}_{w_{ij}}$. It indicates whether the feature word belongs to a subset of feature words *P*_*j*_, *j* = 1, 2, …, *N*, i.e.,

4 $$ {X}_{w_{ij}}=\left\{\begin{array}{c}\ 1,{w}_i\in Q,\\ {}0,{w}_i\notin Q.\end{array}\right. $$

When the word belongs to the feature word set, the Gaussian random distribution is used to fill the added dimension of the character vector $Embedding\_{GAUS}_{w_i}$. Define:

5 $$ {\displaystyle \begin{array}{c} Embeddin{g}_{GAUS_{w_i}}=\frac{\sum_{j=1}^N\left({X}_{w_{ij}}\cdotp Embedding\_{GAUS}_{w_{ij}}\right)}{\sum_{j=1}^N{X}_{w_{ij}}}\\ {}i=1,2,\dots, M,\end{array}}, $$

6 $$ {\displaystyle \begin{array}{c} Embeddin{g}_{GAUS_{w_i}}=\left[{e}_1,{e}_2,\dots, {e}_k\right]\\ {}{e}_m\sim N\left(0,1\right),m=1,2,\dots, k.\end{array}}, $$

Meanwhile, we use zeros to fill the character vector *Embedding* _ *ZERO* of the same dimension when the word does not belong to the feature word set, i.e.,

7 $$ Embedding\_ ZERO=\left[0,0,\dots, 0\right] $$

**Fig. 3:**
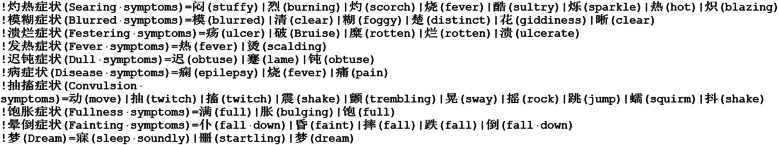
A part of the organized feature words set. We have sorted out different types of feature word sets, and the above are some examples of feature word sets

The examples in the following show the combined of two kinds of character vectors (see Fig. 2) and a part of the organized feature words set (see Fig. 3).

### Dataset annotation

As mentioned in the beginning of the section, we label the TCM clinical terms in the corpus using the “IOB” tags and the category tags marking traditional Chinese medicine, formulas, pattern, symptoms, and disease. The TCM tag set is listed in Table 1.

**Table 1:** There are five types of TCM clinical terms label types shown below

TCM Term Categories	Label Types
Chinese Traditional Medicine	B-MED, I-MED
Formulas	B-FOL, I-FOL
Symptoms	B-SYM, I-SYM
Diseases	B-DES, I-DES
Patterns	B-PAT, I-PAT
Other	O

After the annotation, we use 80% of the dataset as the training set (20,000 words), 10% as the development set (3500 words), and the remaining 10% as the test set (5100 words). In addition, a 100,000-word unlabeled dataset is prepared for semi-supervised learning.

### Semi-supervised training

After the annotation, the Phase 1 model training is run on the annotated training sets by using BiLSTM-CRF. After that, a semi-supervised learning strategy is used, which employs the trained model to extract TCM clinical terms on large scale unlabeled datasets to learn broader data distribution features. We integrate the predicted results with the training set. Then Phase 2 model training is performed on the integrated dataset also using the BiLSTM-CRF model. In these two-stage model trainings, we all use the development set for parameters tuning. Finally, we evaluate our results on the test set using the Phase 2 trained model.

## Results and discussion

The training set for the experiment contains about 20,000 words. The test set contains about 5100 words. The development set contains about 3500 words. There are 1043 TCM clinical terms in training set, 310 in the test set, and 251 in the development set. After the Phase 1 training, the model is used to predict the unlabeled corpus of 100,000 words, and the number of predicted terms is about 5100. The 20,000-word training corpus and the 100,000-word predicted corpus are combined to form a new training corpus, on which we train a model in Phase 2.

Table 2 shows the experimental results of our model. It shows the introduction of feature words and semi-supervised methods help to minimize the annotation. And the value of F1 is enhanced during the extraction. The best result of F1 reaches 78.70%, which can be seen in the Table 2 and Table 3.

**Table 2:** Extraction result for TCM clinical terms in the test dataset

Type	Character Vector	Feature Word Categories	Corpus	Precision	Recall	F1
Supervised	WIKI	0	Train_2w +Dev_3.5 k +Test_5.1 k	0.6831	0.7053	0.6941
Semi-Supervised	WIKI	0	Train_2w_10w +Dev_3.5 k +Test_5.1 k	0.7782	0.7294	0.7530
	TCM	0		0.7474	0.7262	0.7339
	0.3TCM + 0.7WIKI	0		0.7893	0.7294	0.7561
	0.7TCM + 0.3WIKI	0		0.7503	0.7335	0.7298
	0.5TCM + 0.5WIKI	0		0.7935	0.7228	0.7585
	0.5TCM + 0.5WIKI	4		0.7889	0.7525	0.7703
	0.5TCM + 0.5WIKI	39		0.7932	0.7723	07826
	0.5TCM + 0.5WIKI	8		0.7756	0.7987	**0.7870**

**Table 3:** Extraction result for TCM clinical terms in the test dataset

	Precision	Recall	F1
DES	0.7419	0.7667	0.7541
FOL	0.6429	0.5000	0.5625
MED	0.8082	0.8489	0.8281
PAT	0.6250	0.8333	0.7143
SYM	0.7699	0.7909	0.7803
Total	0.7756	0.7987	0.7870

From the above two tables, it is illustrated that the experimental F1 value finally reached 0.787 after performing nine groups of experiments.

We set the first supervised model shown in the Table 2 as the baseline model, which uses the character vectors based on WIKI encyclopedia corpus training as the input layer. The F1 value reached 0.6941. This baseline is the Phase 1 model. After that, we conduct several semi-supervised experiments and experiments based on feature word sets, which belong to the Phase 2 model.

In the semi-supervised learning experiments, the Phase 1 trained model is used to predict the 100,000-word unlabeled data set, then we combine the predicted data with the original labeled training set of 20,000 words to perform Phase 2 model training. The results showed that the F1 value increased to 0.7530 with the semi-supervised learning strategy. We continue the experiment and combine the character vector trained by the WIKI encyclopedia and the TCM related corpus separately, with a custom weight of 0.5. And the combined character vector made a little improvement to the test dataset. The results of semi-supervised experiments show that the data distribution of large-scale unlabeled data contains more abundant data features than the small scale labeled data. The fusion of predicted result of the unlabeled datasets and the labeled datasets facilitate the training of the Phase 2 model to learn a wider range of features distribution, thereby improving the NER extraction result.

In the feature word set experiments, the best “0.5TCM + 0.5WIKI” test result in the above experiment is used as a reference baseline. Therefore, we define 4 categories, 39 categories, and 8 categories of feature words. The experimental results of the 8 types of feature words are the best, and the F1 value increased to 0.7870 (Table 3). Different character vector preprocessing for feature words and non-feature words indirectly emphasizes the importance of feature words in model extraction, so that the extraction model pays more attention to the content before and after the feature words in the training process, which helps to determine the beginning and end boundaries of the entity. And this special treatment eventually improve the sequence labeling result of the model in TCM clinical terms extraction research.

Moreover, it is found that among the five types of TCM clinical terms, the extraction result of MED and SYM is the best, the extraction result of DES and PAT is second, and the effect of FOL is not so good. The reason of this difference resides in two aspects. Firstly, MED, SYM clinical terms relatively have a larger number of occurrences in the training corpus, FOL terms relatively occurs few times. Secondly, the expression of MED and SYM clinical terms have certain regularity. For example, SYM clinical terms show a considerable number of cases ending with a specific character. While in other kinds of terms, this kind of regularity is not very common, such as DES and PAT terms. And FOL terms are the most flexible in the expression. Finally, we find if there are more regular expressions in the TCM clinical terms, the added feature word vector will play a greater role.

## Conclusion

This paper proposes a semi-supervised approach for extracting TCM clinical terms based on character vector. This method is based on the BiLSTM-CRF model, which combines the character vector trained by encyclopedia corpus with that trained by TCM related corpus. Meanwhile, it reduces the cost of manual labeling under the semi-supervised method, and improves the experiment results based on the help of the feature word, which consists of the extraction of TCM clinical terms includes traditional Chinese medicine, symptoms, diseases, patterns, and formulas. The experimental results show that the proposed method can be used for reference in future research. We will continue to explore the effect of using feature words in the extraction of TCM clinical terms and extend to the study of the relation extraction of TCM clinical terms.

## Data Availability

The datasets used and analyzed during the current study are available from the first author upon reasonable requests. The dataset is publically available via https://github.com/JasonWuGenius/TCM_NER.

## Abbreviation

TCM: Traditional Chinese Medicine
